# Disruptive Innovation, Trusted Care

**DOI:** 10.3389/ti.2023.11905

**Published:** 2023-09-04

**Authors:** Gabriel C. Oniscu

**Affiliations:** Division of Transplantation, Department of Clinical Science, Intervention and Technology Karolinska Institute, Stockholm, Sweden

As we emerge from one of the greatest healthcare challenges in human history, we are meeting in Athens for the ESOT Congress 2023 for an opportunity to reconnect and discuss the future directions of travel in organ transplantation and organ replacement.

Despite a significant impact on the delivery of transplant care and a *de facto* research stand still during the last 3 years, we rebounded with renewed energy and it is reassuring to see the extraordinary progress of recent months with a leap in xenotransplantation [1], machine perfusion [2], telemedicine and redefinition of end points in clinical practice and research (Naesens et al.).

The pandemic broke boundaries and brought scientific communities closer in a quest to speed up the discovery of solutions for the challenges we faced. However, it did more than that. It made us reconsider the interaction with our patients and expedited the implementation of novel ways to undertake clinical activity and ensure that we continue to deliver a high quality and trusted care to all patients waiting for or having received a transplant.

The buzz of creativity and collaborative effervescence that defines the ESOT community is demonstrated once again by the innovations presented at the Congress and illustrated by this selection of the top papers submitted.

We live in a world on the brink of radical changes, fueled by an explosion of disruptive innovations that could redesign not only every aspect of our field but our very own way of living. These technologies can bring us closer to a personalized transplant care whilst ensuring a wider reach of transplantation and further improvements in clinical and patient relevant outcomes.

One such technology is *ex situ* machine perfusion. As it gathers clinical momentum, we start to gain a deeper understanding of its effects. Gilbo et al. demonstrated that *ex situ* liver machine perfusion is associated with an unexpected high level of injury to the bile duct, without a clinical translation into a higher incidence of ischaemic biliary strictures. This questions the validity of current assessment criteria and makes an important point that perfusion technologies will completely rehaul the existing concepts of organ assessment and definitions of viability.

Molecular diagnostic technology is another technology that has reached prime time and many of the papers presented at the Congress discuss the benefits and the hurdles of translation into routine care. Giarraputo et al. investigated the molecular refinement of the diagnosis of heart allograft rejection based on whole transcriptome analyses and suggested that a targeted gene panel can be used for detection of antibody mediated rejection and as such can mitigate against some of the challenges that have limited the widespread clinical application of molecular diagnostic technologies to date.

Despite the many advances, there remain persistent challenges in the delivery of transplant care in many parts of the world, of which the lack of organs to meet the growing demand for transplantation and the consequent inequity in access are the top priority. And yet, there is evidence that we can do better. Williment et al. report on a UK initiative that examined the transplant pathways and identified ways to reduce inequity of access, make the best use of available resources and drive innovation in organ transplantation. The authors highlight that a cultural change supported by adequate resources and implementation of technology and decision-making aids is likely to increase organ utilization whilst improving patient experience and outcomes and empowering the transplant community.

One such potential decision making aid is a better understanding of the quality of organ on offer. With an increased proportion of extended criteria donors (ECD), clinicians and patients are sometimes reluctant to take additional risks. Patel et al. demonstrate that ECD kidneys are a valuable resource that provide better outcomes compared to remaining on dialysis and suggest that there is a need for a better definition of extended criteria donors since the current two classifications yield comparable results.

Post pandemic, there remains an acute awareness of other virological challenges. Ushiro-Lamb et al. report on the impact of a strategy for hepatitis E virus screening in the UK. Universal donor screening has ensured that patients at risk are identified and managed early to minimize the risks to the transplant and ensure a judicious use of available organs. Whilst minimizing the risk of viral transmission. Along the same lines, Böhler et al. provide a 6 year report on the risk of donor viral and malignancy transmission in Germany highlighting the importance of a reliable evaluation and alert system to assess the risks, assess the decision making and improved the safety of transplantation.

Whilst transplant is the desired treatment option for all patients with end organ failure, this may not be attainable for many reasons. As such, part of the shared decision making process, alternative treatment options, with their pros and cons should be fully discussed, in order to create and maintain a trusted care environment. Francica et al. illustrate the importance of discussing alternative treatment options for heart failure in the context of changes in technology and integration with the overall transplant strategy.

Disruptive innovation and trusted care are core values for the ESOT community and it is incumbent on us to ensure that all these innovations and efforts to increase organ utilization supported by evidence based decisions are integrated in clinical care across Europe and that we share this knowledge to widen access to transplantation.

This collection of manuscripts demonstrates yet again the high standard of science and research presented at the ESOT Congress 2023 and provides the reassurance that we are in a very strong position to continue innovating and deliver the high quality care expected by our patients.
